# The temporal relationship between the neural and vascular actions of kallidin within the nose

**DOI:** 10.1155/S0962935193000298

**Published:** 1993

**Authors:** K. Rajakulasingam, L. C. K. Lau, R. Polosa, M. K. Church, S. T. Holgate, P. H. Howarth

**Affiliations:** Immunopharmacology Group Centre Block Southampton General Hospital Tremona Road, Southampton SO9 4XY UK

## Abstract

The time course of effect of the B_2_-receptor agonist kallidin (K) on induced changes of nasal airflow, rhinorrhoea, nasal pain, sneezing and nasal microvascular leakage has been examined and compared with its B_2_ metabolite agonist bradykinin (B) and the B_1_-agonist [des-arg^9^]-bradykinin (D). When administered as a single dose K and B induced an immediate sensation of pain, rhinorrhoea, elevations in lavage albumin and protein levels and a sustained increase in nasal airways resistance (NAR) for 5–40 min post-challenge. [des-arg^9^]-Bradykinin and vehicle placebo (V) were without effect on any of these indices. These studies identify the action of K and B within the nose and differentiate the neural and vascular effects of these kinins in addition to suggesting the potential that nasal blockage and nasal microvascular leakage represent alterations in differing vascular compartments. These findings have implications for the understanding and therapeutic manipulation of rhinitis.


Mediators of Inflammation 2, 217–223 (1993)

### The temporal relationship between the neural and vascular actions of kallidin within the nose

K. Rajakulasingam,CA L. C. K. Lau, R. Polosa, M. K. Church, S. T. Holgate and P. H. Howarth

Immunopharmacology Group, Centre Block, Southampton General Hospital, Tremona Road, Southampton SO9 4XY, UK

CA Corresponding Author

**THE time course of effect of the $B_2$-receptor agonist kallidin (K) on induced changes of nasal airflow, rhinorrhoea, nasal pain, sneezing and nasal microvascular leakage has been examined and compared with its $B_2$ metabolite agonist bradykinin (B) and the $B_1$-agonist [des-arg$^9$]-bradykinin (D). When administered as a single dose K and B induced an immediate sensation of pain, rhinorrhoea, elevations in lavage albumin and protein levels and a sustained increase in nasal airways resistance (NAR) for 5–40 min post-challenge. [des-arg$^9$]-Bradykinin and vehicle placebo (V) were without effect on any of these indices. These studies identify the action of K and B within the nose and differentiate the neural and vascular effects of these kinins in addition to suggesting the potential that nasal blockage and nasal microvascular leakage represent alterations in differing vascular compartments. These findings have implications for the understanding and therapeutic manipulation of rhinitis.**

**Key words:** Bradykinin, [des-arg$^9$]-Bradykinin, Kallidin

### Introduction

The introduction of potent and specific $H_1$-antihistamines in the 1940s, confirmed histamine as an important mediator of allergic rhinitis but, in producing only partial relief of symptoms, revealed the participation of other mediators in this disease. Among these are the kinins, including, bradykinin and kallidin (lysyl bradykinin), both of which are potent vasoactive peptides formed as cleavage products from the action of kallikreins on high and low molecular weight kininogens respectively.[1] Metabolism of bradykinin occurs with cleavage of the N-terminal [des-arg$^9$] residue to a peptide which is reported to be active on the $B_1$-receptor subtype.[2]

The presence of increased kinin recovery by nasal lavage during the early[3] and late[4] phases of allergen-provoked rhinitis and also in naturally occurring rhinitis[5] provides evidence that kinin generating systems are operative in this disease and their involvement in symptom generation is suggested from nasal challenge studies. Nasal insufflation with bradykinin elicits many of the clinical features of rhinitis including nasal blockage, rhinorrhoea and increased micro-vascular leakage.[6,7] Less is known, however, of the nasal effects of kallidin.

Kallidin comprises approximately half of the amount of kinin recovered following nasal allergen challenge[3] and accords with the finding of increased tissue kallikrein in lavage fluid.[8] Concordant with this, elevated levels of aminopeptidase, capable of converting kallidin to bradykinin, have been demonstrated in post-allergen challenge nasal lavage.[9] Bradykinin and kallidin both produce nasal blockage by interacting with the $B_2$-receptor subtype and are of comparable potencies when given in incremental doses.[7] However, in the lower airways, kallidin is about three times less potent as a bronchoconstrictor than bradykinin in molar terms.[10]

To further explore the comparative range of effects of kallidin within the nose, the time course of action of this kinin on individual nasal symptoms and signs has been studied in parallel with comparable amounts of the nasal effects of the $B_2$ agonist bradykinin and the $B_1$ agonist [des-arg$^9$]-bradykinin in healthy volunteers.

### Subjects and Methods

The study was conducted in eight non-rhinitic, non-atopic healthy volunteers (six males and two females) with a mean age of 29 years. All subjects refrained from taking any medications throughout the study period. None of the subjects had a history of recent upper respiratory tract infection, nasal polyps, infective rhinitis, nasal surgery or nasal deformities and all gave written informed consent. The study was approved by the Southampton hospitals and University joint ethical subcommittee. The study was conducted in three phases.

*Phase I:* Subjects attended on four occasions at the same time of the day to receive nasal challenge with incremental doses of either bradykinin, kallidin, [des-arg$^9$]-bradykinin or vehicle placebo in a double-blind cross-over manner. The objective response parameter was nasal airways resistance (NAR). At each visit, subjects were rested for 15

© 1993 Rapid Communications of Oxford Ltd min and baseline measurements of NAR were made by active posterior rhinomanometry using a Mercury NR6 Rhinomanometer (Mercury instruments, Glasgow, Scotland) as described previously.[7] The measurement of NAR has a coefficient of variation of repeated measurements of 10.7%. Providing the baseline values of NAR did not vary by > 10%, subjects then underwent nasal challenge with vehicle followed by incremental doses of either bradykinin, kallidin, [des-arg$^9$]-bradykinin or vehicle placebo at 20 min intervals until the top challenge dose had been achieved. The three kinins (Nova Biochem Ltd, Nottingham, UK) were dissolved in ethanol (10%) and 0.9% sodium chloride to produce concentrations of 0.077, 0.77, 3.85 and 7.7 mg/ml. These doses were chosen from previous studies.[6,7] The purity of the synthetic kinins was confirmed by high-performance liquid chromatography (HPLC) as described previously.[7] Nasal challenge was undertaken bilaterally using a hand-held pump spray delivering 0.13 ml per activation with a coefficient of variation of output of 8.4%. The spray was placed in one nostril while occluding the other and activated once during quiet inspiration. The procedure was then repeated in the opposite nostril. At the concentrations employed, this method delivered total kinin doses of 20, 200, 1 000 and 2 000 μg equally divided between the two nostrils. After each challenge repeat measurements of NAR were made at 3, 5, 10, 15 and 20 min post-challenge.

*Phase 2:* To investigate the time course of effect of nasal insufflation with kinins on induced symptoms and signs, each of the eight subjects attended at the same time of the day on four further occasions separated by at least 7 days to receive nasal challenge with a single dose (2 000 μg) of bradykinin, kallidin, [des-arg$^9$]-bradykinin or vehicle in a double-blind crossover manner. Repeat measurements of NAR were made at 3, 5, 10, 15, 20, 25, 30, 35 and 40 min post-challenge. Subjects also recorded their symptom of nasal pain/discomfort on a 10 cm visual analogue scale, before and at 3, 5, 10, 15, 20, 25, 30, 35 and 40 min post-challenge. Rhinorrhoea was measured 45 s before each of these time points by asking the subjects to blow secretions into a pre-weighed tissue towel. The tissue towels were then immediately reweighed and the weight of secretion calculated by subtraction. The symptom of sore throat was recorded as present or absent and the number of sneezes following challenge was counted.

*Phase 3:* To investigate the time-course of action of these kinins on nasal vascular permeability, each of the eight subjects attended on an additional four occasions, and were randomly allocated to receive nasal challenge with the same single dose of bradykinin, kallidin, [des-arg$^9$]-bradykinin or vehicle as for phase 2, in a double-blind, cross-over study design. At each attendance sequential nasal lavage was performed bilaterally five times, at 1-min intervals, prior to nasal insufflation challenge. Subjects were instructed to tilt their heads backwards about 30° from the horizontal while in the sitting position, to hold their breath and refrain from swallowing. Two and a half ml of 0.9% prewarmed saline was instilled into each nostril and after 10 s subjects flexed their necks and expelled the mixture of airways surface liquid and saline into a collection vessel. Nasal lavage was then repeated at 3, 5, 10, 15, 20, 25, 30, 35 and 40 min after each of the nasal challenges.

*Analysis of nasal lavage fluid:* Lavage samples were immediately stored on ice until the conclusion of the experiments when they were frozen at −20°C to await protein analysis. After thawing at room temperature, total protein concentrations were measured in 0.25 ml replicates by the method given by Bradford[11] using Coomassie Blue G-250 reagent (Pierce, Rockford, IL, USA) as indicator. Absorbance read at 595 nm was compared with a standard curve constructed with bovine serum albumin (Sigma, Poole, UK) to give absolute protein concentrations.

Albumin concentrations were measured by rocket immunoelectrophoresis.[12] Three microlitre aliquots of nasal lavage fluid were introduced into wells cut in agarose gel and subjected to electrophoresis (4 V/cm) for 20 h into a 'window' of agarose containing 1.18 μl/cm$^2$ of rabbit anti-human albumin antibody (Nordic, Tilburg, Netherlands). Immunoprecipitates were stained with Coomassie Brilliant Blue. Albumin levels were quantified by comparison with standard curves constructed using human serum albumin (Sigma, Poole, UK). The sensitivity of the albumin assay was 1 μg/ml and the coefficient of variation of repeated measurements is 5%.

*Data analysis:* On the different challenge days baseline measurements of NAR, lavage albumin and total protein were compared using the Friedman's two-way ANOVA test. The time-dependent changes in NAR (Pa/cm$^3$s), lavage albumin and total protein were compared both within groups and between groups using Friedman's test. To assess differences between treatments, the mean area under the curve (AUC) for each time period of observation was calculated and compared using the Wilcoxon's signed rank test for paired data. The Wilcoxon's signed rank test was also used for comparisons of the challenges on the quantity of nasal secretions, the number of sneezes and the visual analogue scores for nasal pain. Values are expressed as mean ± standard error of mean or median values with ranges.

### Results

*Phase 1:* There were no significant differences between the baseline NAR measurements in the eight subjects on any of the four challenge days ($p > 0.05$). Both bradykinin and kallidin caused a dose-dependent increase in NAR ($p < 0.01$), while [des-arg$^9$]-bradykinin and vehicle were without significant effect (Fig. 1). There was no significant difference in the effects of bradykinin and kallidin on NAR at any dose level ($p > 0.05$) whether assessed as peak response or area under the curve.

*Phase 2:* There were no significant differences between baseline NAR measurements in the eight subjects on any of the four challenge days ($p > 0.05$). Insufflation of vehicle had no significant effect on NAR. Bradykinin administered as a single dose of 2000 μg caused a rapid increase in NAR reaching maximum of 71.4% above baseline at 15 min ($p < 0.05$) and gradually declining over 40 min to 50.0% above baseline (Fig. 2). Kallidin also increased NAR by a median maximum of 57.1% ($p < 0.05$). This peak was reached 20 min post-challenge. The increase in NAR with challenge was significantly greater for bradykinin than kallidin at 5 and 15 min post-challenge but thereafter there was no significant difference from that seen with bradykinin (Fig. 2). However, there was no significant difference between the areas under the time–response curves for both agonists ($p > 0.05$). The increase in NAR was significantly greater than the pre-challenge measurements from 3 to 40 min ($p < 0.05$). In contrast [deg-arg$^9$]-bradykinin produced no significant change in NAR.

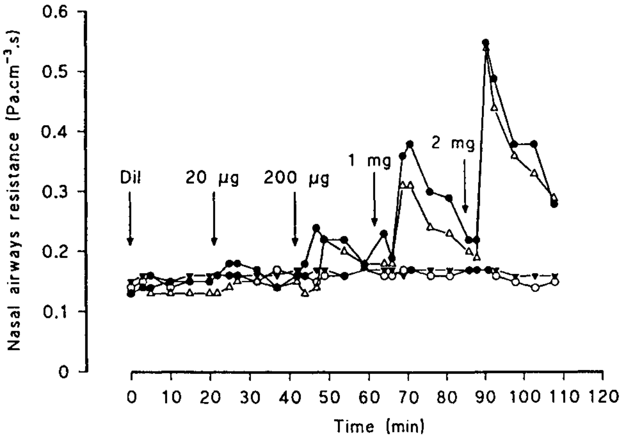

FIG. 1. Dose-dependent changes in NAR following nasal challenge with incremental doses (20, 200, 1 000 and 2 000 μg) of bradykinin (●), kallidin (△), [des-arg$^9$]-bradykinin (▼) and 10% ethanol saline (○). Each result is the median of observations in eight subjects.

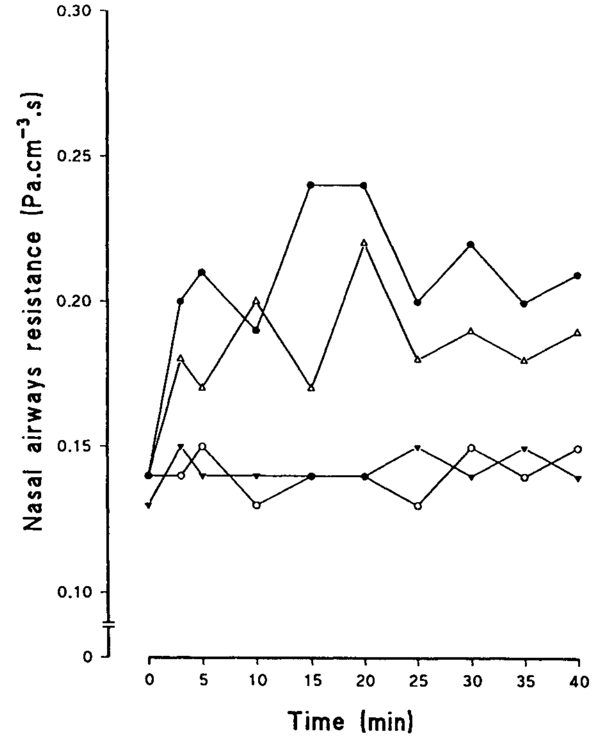

FIG. 2. NAR changes following nasal challenge with single dose of bradykinin 2 000 μg (●), kallidin 2 000 μg (△), [des-arg$^9$]-bradykinin 2 000 μg (▼) and 10% ethanol saline (○). Each result is the median of observations in eight subjects.

When compared with vehicle, bradykinin and kallidin, but not [des-arg$^9$]-bradykinin, produced rhinorrhoea, with median total secretion weights of 376 (range 179–597) mg and 330 (range 60–1351) mg, respectively ($p < 0.05$). The increase in nasal secretions was maximum 3 min post-challenge and declined rapidly to very low levels, indistinguishable from placebo, by 20 min post-challenge (Fig. 3A). Both bradykinin and kallidin produced similar degrees of nasal pain, with median (range) values of 3.45 (range 0.6–7.2) cm and 3.4 (range 0–7.5) cm, respectively ($p < 0.05$). Nasal pain reached maximum at 3 min post-challenge and lasted for 5 min (Fig. 3B). Neither [des-arg$^9$]-bradykinin nor vehicle produced algesic effect (Fig. 3B). None of the subjects complained of nasal itch and following bradykinin and kallidin challenge three subjects complained of sore throat lasting up to 25 min. None of the subjects sneezed following kinin nasal challenge.

*Phase 3:* There were no significant difference between the fifth baseline nasal lavage albumin levels in the eight subjects on the four challenge days, the group means being 24.73 ± 5.71, 26.14 ± 10.1, 27.71 ± 12.9 and 27.7 ± 9.14 μg/ml for subjects receiving bradykinin, kallidin, [des-arg$^9$]-bradykinin and vehicle, respectively. Insufflation with bradykinin and kallidin increased the concentration of albumin recovered in the nasal

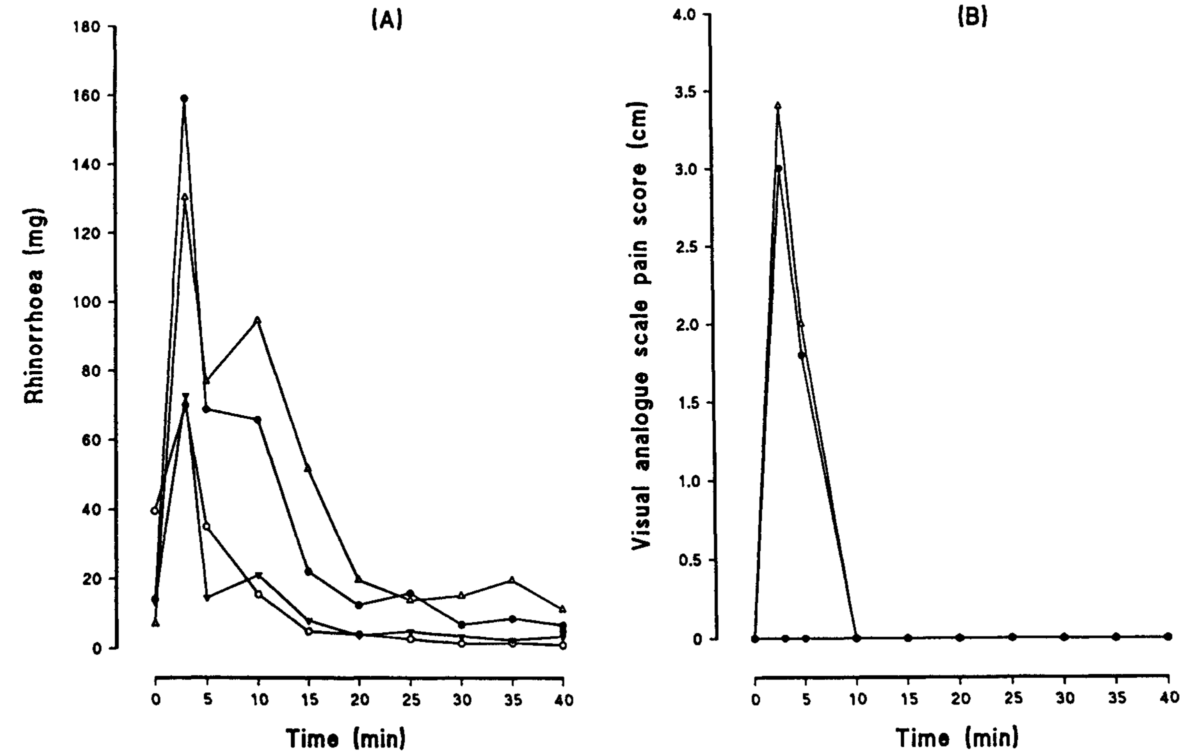

FIG. 3. Effects of bradykinin 2 000 μg (●), kallidin 2 000 μg (△), [des-arg$^9$]-bradykinin 2 000 μg (▼) and 10% ethanol saline vehicle (○) on (A) rhinorrhoea, (B) nasal pain. Each result is the median of observations in eight subjects.

lavage ($p < 0.05$) (Fig. 4A). On repeated lavage, maximum albumin concentration achieved with both challenges occurred within 10 min and decayed progressively to baseline by 40 min (Fig. 4A). By contrast nasal challenge with [des-arg$^9$]-bradykinin and vehicle failed to induce significant changes in lavage albumin concentration (Fig. 4A).

There were no significant differences between the fifth baseline nasal lavage total protein levels in the eight subjects on the four challenge days, the group

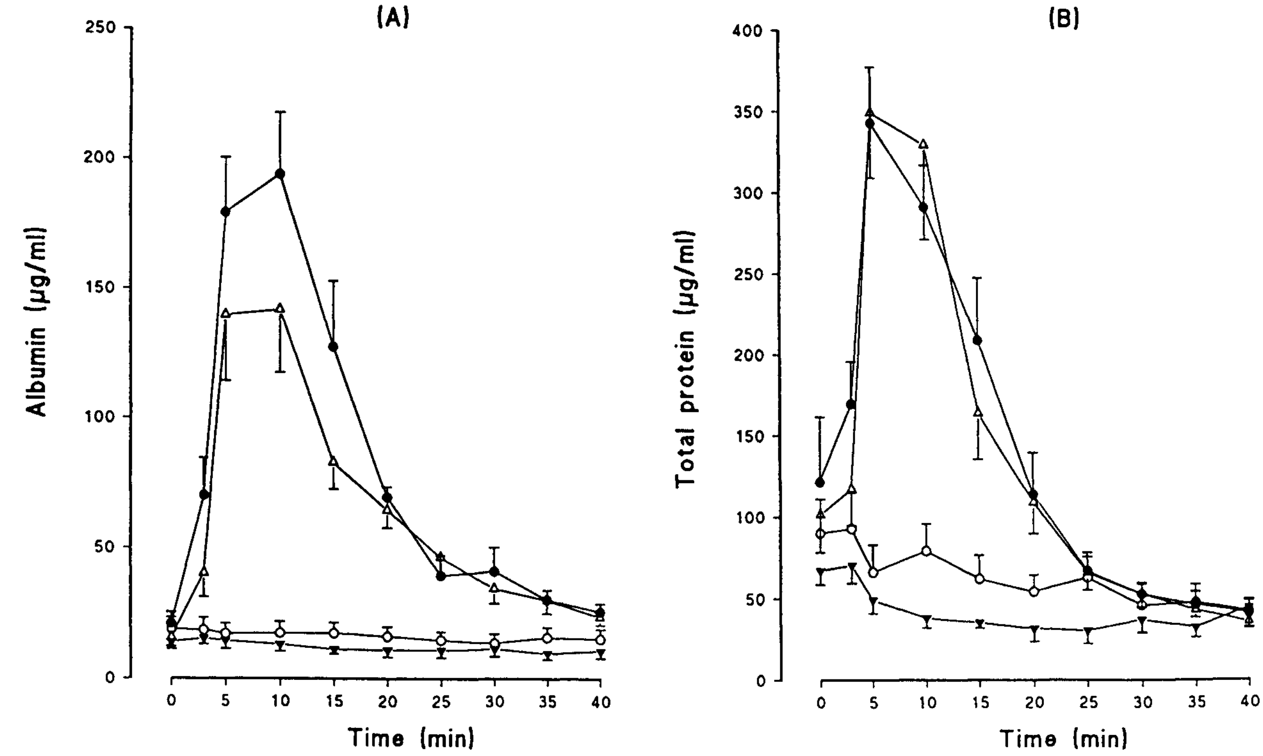

FIG. 4. Nasal lavage (A) albumin and (B) total protein changes following nasal challenge with single dose of bradykinin 2 000 μg (●), kallidin 2 000 μg (△), [des-arg$^9$]-bradykinin 2 000 μg (▼) and 10% ethanol saline (○). Each result is the mean ± S.E. of mean of observations in eight subjects.

means being 121.58 ± 40.14, 101.54 ± 23.12, 67.25 ± 8.63 and 90.25 ± 20.88 $\mu$g/ml for subjects receiving bradykinin, kallidin, [des-arg$^9$]-bradykinin and vehicle, respectively. When compared to vehicle, both bradykinin and kallidin induced significant increases in lavage total protein levels ($p < 0.05$) with a time course similar to that seen with albumin whereas [deg-arg$^9$]-bradykinin had no effect (Fig. 4B). The albumin and total protein responses following bradykinin and kallidin were not significantly different when compared using the AUC ($p > 0.05$).

### Discussion

This study extends and confirms our previous findings that $B_2$-agonists bradykinin and kallidin induce a dose-dependent increase in nasal airways resistance while the $B_1$-agonist [des-arg$^9$]-bradykinin is without effect.[7] By selecting equi-doses of kallidin and bradykinin it has been possible to further explore their time course of action and to investigate their relative effects not only on nasal blockage but also on nasal pain, sneezing, rhinorrhoea and on increments in nasal lavage albumin and total protein levels. Both kallidin and bradykinin but not [des-arg$^9$]-bradykinin induced changes in all these indices with the exception of sneezing.

Within human lower airways kallidin is three times less potent than bradykinin as a bronchoconstrictor when these kinins are compared on a molar basis.[10] In contrast we have previously found that kallidin and bradykinin, when administered in incremental doses, to be approximately equipotent in the nasal obstructive effect within the upper airways.[7] Similar differences have been reported in other species and our finding is not inconsistent with other studies where bradykinin and kallidin have been shown to have varying potency of action.[13,14] In rats, kallidin has been shown to be more potent than bradykinin in causing relaxation of isolated smooth muscle preparation from duodenum, although on isolated smooth muscles from guinea-pig ileum and rat uterus a reversed order of potency has been observed.[13] Kallidin is also more potent than bradykinin as a local vasodilator in dogs.[14] In the canine tracheal epithelium, which is enriched in kinin receptors, kallidin is equipotent with bradykinin in inducing chloride secretion.[15] The relevance of such a consideration to rhinitis is the identification that kallidin represents 45% of the total kinin pool in lavage fluid following nasal allergen challenge.[3] Single dose nasal administration of both kallidin and bradykinin but not [des-arg$^9$]-brady-kinin induced a significant increase in nasal airways resistance. For each of the $B_2$-receptor agonists this was significant from 5 to 40 min post-challenge (Fig. 2). Although the effect of bradykinin appears greater than that of kallidin there was no significant difference between the areas under the time–response curves for both agonists. However, when compared at individual time points, the NAR increase following bradykinin nasal challenge was significantly greater at 5 and 15 min post-challenge. The peak NAR responses following kallidin and bradykinin were at 20 and 15 min post-challenge, respectively.

The present study also identifies that both kallidin and bradykinin induce nasal pain, rhinorrhoea, nasal blockage and an increase in nasal lavage protein levels. These are the first human studies investigating in detail these neural and vascular mediated effects of kallidin within the nose. The nasal blockage and increase in lavage protein levels are considered to be manifestations of direct vascular actions. Inconsistent with this, kinin $B_2$-receptors have been localized to the vasculature of the nasal mucosa,[16] and the nasal obstructive response, but not the plasma protein exudation, has been shown to be prevented by prior administration of the $\alpha$-adrenergic agonist oxymetazoline.[17] It is therefore likely that nasal obstruction and plasma protein extravasation represent different vascular mechanisms. Consistent with this, the time course of increase in lavage protein levels which peaks at 10 min and resolves within 20 min post-challenge differs from the time course of effect on nasal airflow, further suggesting that the nasal blockage and plasma protein leakage are two independent vascular effects (Figs 2 and 4). Nasal obstruction arises from engorgement of venous plexi within the turbinates.[18] These plexi are under sympathetic regulation and factors which increase sympathetic stimulation (i.e., exercise, $\alpha$-adrenergic agonists) increase nasal airflow and reduce nasal airways resistance whereas mediators, such as histamine, prostaglandins and kinins which have direct vasodilator properties induce nasal obstruction.

In addition, laser doppler flowmetry studies have identified changes in blood flow in the superficial mucosal vasculature following bradykinin challenge (Rajakulasingam K, unpublished findings). This is not always in concordance with the nasal obstructive response and it is likely that endothelial contraction with opening of gap junctions in post-capillary venules present beneath the basement membrane and around glands in the nasal mucosa, are responsible for the increase in lavage protein levels.[19] Consistent with this, changes in vascular permeability produce unrestricted plasma molecular leak, concordant with the albumin and total protein increments in lavage with kallidin and bradykinin. Although it is not possible to exclude glandular secretion of protein, this is less likely as our study shows that the predominant protein in nasal lavage is albumin (Fig. 4), a plasma protein synthesized by liver and no $B_2$-receptors have been localized to glandular structures within the human nose.[16] The neural effects of kinin insufflation are pain and possibly rhinorrhoea. With histamine nasal insufflation the rhinorrhoea is bilateral following unilateral challenge and can be inhibited by antimuscarinic pretreatment indicative of a reflex vagal mechanism.[20]

In a separate investigation we have been unable to identify any protective effect of nasal ipratropium bromide on bradykinin induced rhinorrhoea.[21] This suggests that the mechanism of rhinorrhoea with bradykinin differs from histamine. Consistent with this the sensory neural stimulation induced by kallidin and bradykinin differs from histamine is not inducing sneezing. Thus the nasal pain induced by kinins must reflect different sensory neural pathways than those stimulated by histamine. That these nerves are distinct from the classical unmyelinated 'C' sensory nerves is further suggested by the identification that the ipsilateral secretory effects of unilateral capsaicin nasal challenge are blocked by the coadministration of intramuscular atropine and topical ipratropium bromide[22] and that repeated nasal capsaicin pretreatment has failed to diminish the algesic response to kallidin.[23] This indicates that the human nose differs from animals in its response to kinins, as the effects of bradykinin in canine and rat airways have been shown to be mediated via capsaicin sensitive, sensory neurones,[24,25] and further highlights the interspecies differences in response to kinins.

It is possible that the rhinorrhoea induced by kallidin and bradykinin which peaks at the same time as the nasal pain following challenge (Fig. 3) represents stimulation of neuropeptide containing nerves with consequent peptide release and glandular secretion. Alternatively, as bradykinin has been reported to stimulate prostaglandin synthesis[26,27] it is possible that this could account for the nociceptive and secretory effects of kallidin and bradykinin. Against such a possibility is the rapidity of onset of the algesic effect, and the recent report that oral acetyl salicylic acid had no effect on the nasal discomfort following nasal bradykinin challenge.[17] A further possibility is that the rhinorrhoea is a reflection of plasma leakage rather than glandular secretion. While studies in cats and ferrets have indicated that apparent secretion induced by bradykinin is not associated with an increase in mucous glycoconjucates[16] the rapid increase in rhinorrhoea observed in this study, which occurred before marked increases in lavage albumin were observed, would argue against such a possibility. Thus the exact mechanism of the rhinorrhoea remains to be elucidated.

In conclusion this study draws attention to the vascular actions of kallidin and bradykinin within the nose and indicates that obstruction and vascular permeability represent distinct entities. The prolonged action of these kinins on nasal obstruction suggests their importance in clinical disease. However, the contribution of kinins to the symptomatology of rhinitis will not be fully elucidated until potent and selective inhibitors of kinin generation or antagonists of kinin receptor mediated effects become available.

### References

1. Proud D. Kinin formation: mechanisms and role in inflammatory disorders. *Annu Rev Immunol* 1988; **6:** 49–83.
2. Regoli D, Barabe J. Pharmacology of bradykinin and related kinins. *Pharmacol Rev* 1980; **32:** 1–46.
3. Proud D, Togias A, Naclerio RM, Crush SA, Norman PS, Lichtenstein LM. Kinins are generated *in vivo* following nasal airway challenge of allergic individuals with allergen. *J Clin Invest* 1983; **72:** 1678–1685.
4. Naclerio RM, Proud D, Togias AG, *et al.* Inflammatory mediators in late antigen-induced rhinitis. *N Engl J Med* 1985; **313:** 65–70.
5. Svennsson C, Andersson M, Persson CGA, Venge P, Alkner U, Pipkorn U. Albumin, bradykinin and eosinophil cationic protein on the nasal mucosal surface in patients with hay fever during natural allergen exposure. *J Allergy Clin Immunol* 1990; **85:** 828–833.
6. Proud D, Reynolds CJ, Lacapra S, Sobotka AK, Lichtenstein LM, Naclerio RM. Nasal provocation with bradykinin induces symptoms of rhinitis and a sore throat. *Am Rev Respir Dis* 1988; **137:** 613–616.
7. Rajakulasingam K, Polosa R, Holgate ST, Howarth PH. Comparative nasal effects of bradykinin, kallidin and [des-arg$^9$]-bradykinin in atopic rhinitis and normal volunteers. *J Physiol* 1991; **437:** 577–587.
8. Baumgarten CR, Nichols RC, Naclerio RM, Proud D. Concentrations of glandular kallikrein in human nasal secretions increase during experimentally-induced allergic rhinitis. *J Immunol* 1986; **137:** 1323–1328.
9. Proud D, Baumgarten CR, Naclerio RM, Ward PE. Kinin metabolism in nasal secretions during experimentally-induced allergic rhinitis. *J Immunol* 1987; **138:** 428–434.
10. Polosa R, Holgate ST. Comparative airway response to inhaled bradykinin, kallidin and [des-arg$^9$]-bradykinin in normal and asthmatic subjects. *Am Rev Respir Dis* 1990; **142:** 1367–1371.
11. Bradford MM. A rapid and sensitive method for the quantitation of microgram quantities of protein utilizing the principle of protein–dye binding. *Anal Biochem* 1976; **72:** 248.
12. Weeke B. Rocket immunoelectrophoresis. *Scand J Immunol* 1973; **1:** 37–46.
13. Reis ML, Okino L, Silva MR. Comparative pharmacological actions of bradykinin and related kinins of larger molecular weights. *Biochem Pharmacol* 1971; **20:** 2935–2946.
14. McCarthy DA, Potter DE, Nicolaides ED. An *in vivo* estimation of the potencies and half-lives of synthetic bradykinin and kallidin. *J Pharmacol Exp Therap* 1965; **148:** 117–122.
15. Rangachari PK, McWade D, Donoff B. Luminal receptors for bradykinin on the canine tracheal epithelium: functional subtyping. *Regul Peptides* 1988; **21:** 237–244.
16. Baraniuk J, Lundgren JD, Mizoguchi H, *et al.* Bradykinin and respiratory mucous membranes. *Am Rev Respir Dis* 1990; **141:** 706–714.
17. Churchill L, Pongracie JA, Reynolds CJ, Naclerio RM, Proud D. Pharmacology of nasal provocation with bradykinin: studies of tachyphylaxis, cyclooxygenase inhibition, α-adrenergic stimulation and receptor subtype. *Int Arch Allergy Appl Immunol* 1991; **95(4):** 322–331.
18. Eccles R. Neurological and pharmacological considerations. In: Proctor DR and IB Anderden, eds. *The Nose: Upper Airway Physiology and the Atmospheric Environment.* Amsterdam: Elsevier, 1982; 191–214.
19. Cauna N, Hinderer KH. Fine structure of blood vessels of the human nasal respiratory mucosa. *Ann Otol Rhinol Laryngol* 1969; **78:** 865–879.
20. Mygind N, Secher C, Kirkegaard J. Role of histamine and antihistamines in the nose. *Eur Respir J* 1983; **64** (suppl 128): 16–20.
21. Rajakulasingam K, Polosa R, Lau LCK, Church MK, Holgate ST, Howarth PH. The influence of terfenadine and ipratropium bromide alone and in combination on bradykinin induced nasal symptoms and plasma protein leakage. *Clin Exp Allergy* 1992; **22:** 717–723.
22. Stjarne P, Lundblad L, Lundberg JM, Anggard A. Capsaicin and nicotine-sensitive afferent neurones and nasal secretion in healthy human volunteers and in patients with vasomotor rhinitis. *Br J Pharmacol* 1989; **96:** 693–701.

23. Geppetti P, Fusco BF, Alessandri M, *et al.* Kallidin applied to the human nasal mucosa produces algesic response not blocked by capsaicin densensitization. *Regulatory Peptides* 1991; **33**: 321–329.
24. Kaufman MP, Coleridge HM, Coloeridge JCG, Baker DG. Bradykinin stimulates afferent vagal C-fibres in intrapulmonary airways in dogs. *J Appl Physiol* 1980; **48**: 511–517.
25. Lundberg JM, Saria A. Capsaicin induced desensitization of the airway mucosa to cigarette smoke, mechanical and chemical irritants. *Nature* (*Lond*) 1983; **302**: 251–253.
26. Leikauf GD, Ueki IF, Nadel JA, Widdicombe JH. Bradykinin stimulates chloride secretion and prostaglandin $E_2$ release by canine tracheal epithelium. *Am J Physiol* 1985; **248**: F48–55.
27. Conklin BR, Burch RM, Steranka LR, Axelrod J. Distinct bradykinin receptors mediate stimulation of prostaglandin synthesis by endothelial cells and fibroblasts. *J Pharmacol Exp Therap* 1988; **244**: 646–649.

ACKNOWLEDGEMENTS. We are grateful to Dr Andrew Walls for his technical advice on protein assay and Dr Doreen Ashworth (Ferring Institute) for HPLC analysis of the purity of kinins. This study was supported by a grant from the Ferring Peptide Research Partnership, Malmo, Sweden.

**Received 23 February 1993; accepted in revised form 16 March 1993**

## References

[PDF_00606] Baraniuk J. N., Lundgren J. D., Mizoguchi H., Peden D., Gawin A., Merida M., Shelhamer J. H., Kaliner M. A. (1990). Bradykinin and respiratory mucous membranes. Analysis of bradykinin binding site distribution and secretory responses in vitro and in vivo.. Am Rev Respir Dis.

[PDF_00593] Bradford M. M. (1976). A rapid and sensitive method for the quantitation of microgram quantities of protein utilizing the principle of protein-dye binding.. Anal Biochem.

[PDF_00615] Cauna N., Hinderer K. H. (1969). Fine structure of blood vessels of the human nasal respiratory mucosa.. Ann Otol Rhinol Laryngol.

[PDF_00608] Churchill L., Pongracic J. A., Reynolds C. J., Naclerio R. M., Proud D. (1991). Pharmacology of nasal provocation with bradykinin: studies of tachyphylaxis, cyclooxygenase inhibition, alpha-adrenergic stimulation, and receptor subtype.. Int Arch Allergy Appl Immunol.

[PDF_00640] Conklin B. R., Burch R. M., Steranka L. R., Axelrod J. (1988). Distinct bradykinin receptors mediate stimulation of prostaglandin synthesis by endothelial cells and fibroblasts.. J Pharmacol Exp Ther.

[PDF_00628] Geppetti P., Fusco B. M., Alessandri M., Tramontana M., Maggi C. A., Drapeau G., Fanciullacci M., Regoli D. (1991). Kallidin applied to the human nasal mucosa produces algesic response not blocked by capsaicin desensitization.. Regul Pept.

[PDF_00631] Kaufman M. P., Coleridge H. M., Coleridge J. C., Baker D. G. (1980). Bradykinin stimulates afferent vagal C-fibers in intrapulmonary airways of dogs.. J Appl Physiol Respir Environ Exerc Physiol.

[PDF_00637] Leikauf G. D., Ueki I. F., Nadel J. A., Widdicombe J. H. (1985). Bradykinin stimulates Cl secretion and prostaglandin E2 release by canine tracheal epithelium.. Am J Physiol.

[PDF_00634] Lundberg J. M., Saria A. (1983). Capsaicin-induced desensitization of airway mucosa to cigarette smoke, mechanical and chemical irritants.. Nature.

[PDF_00600] MCCARTHY D. A., POTTER D. E., NICOLAIDES E. D. (1965). AN IN VIVO ESTIMATION OF THE POTENCIES AND HALF-LIVES OF SYNTHETIC BRADYKININ AND KALLIDIN.. J Pharmacol Exp Ther.

[PDF_00617] Mygind N., Secher C., Kirkegaard J. (1983). Role of histamine and antihistamines in the nose.. Eur J Respir Dis Suppl.

[PDF_00572] Naclerio R. M., Proud D., Togias A. G., Adkinson N. F., Meyers D. A., Kagey-Sobotka A., Plaut M., Norman P. S., Lichtenstein L. M. (1985). Inflammatory mediators in late antigen-induced rhinitis.. N Engl J Med.

[PDF_00590] Polosa R., Holgate S. T. (1990). Comparative airway response to inhaled bradykinin, kallidin, and [des-Arg9]bradykinin in normal and asthmatic subjects.. Am Rev Respir Dis.

[PDF_00584] Proud D., Baumgarten C. R., Naclerio R. M., Ward P. E. (1987). Kinin metabolism in human nasal secretions during experimentally induced allergic rhinitis.. J Immunol.

[PDF_00565] Proud D., Kaplan A. P. (1988). Kinin formation: mechanisms and role in inflammatory disorders.. Annu Rev Immunol.

[PDF_00578] Proud D., Reynolds C. J., Lacapra S., Kagey-Sobotka A., Lichtenstein L. M., Naclerio R. M. (1988). Nasal provocation with bradykinin induces symptoms of rhinitis and a sore throat.. Am Rev Respir Dis.

[PDF_00569] Proud D., Togias A., Naclerio R. M., Crush S. A., Norman P. S., Lichtenstein L. M. (1983). Kinins are generated in vivo following nasal airway challenge of allergic individuals with allergen.. J Clin Invest.

[PDF_00581] Rajakulasingam K., Polosa R., Holgate S. T., Howarth P. H. (1991). Comparative nasal effects of bradykinin, kallidin and [Des-Arg9]-bradykinin in atopic rhinitic and normal volunteers.. J Physiol.

[PDF_00603] Rangachari P. K., McWade D., Donoff B. (1988). Luminal receptors for bradykinin on the canine tracheal epithelium: functional subtyping.. Regul Pept.

[PDF_00567] Regoli D., Barabé J. (1980). Pharmacology of bradykinin and related kinins.. Pharmacol Rev.

[PDF_00597] Reis M. L., Okino L., Rocha e Silva M. (1971). Comparative pharmacological actions of bradykinin and related kinins of larger molecular weights.. Biochem Pharmacol.

[PDF_00623] Stjärne P., Lundblad L., Lundberg J. M., Anggård A. (1989). Capsaicin and nicotine-sensitive afferent neurones and nasal secretion in healthy human volunteers and in patients with vasomotor rhinitis.. Br J Pharmacol.

[PDF_00574] Svensson C., Andersson M., Persson C. G., Venge P., Alkner U., Pipkorn U. (1990). Albumin, bradykinins, and eosinophil cationic protein on the nasal mucosal surface in patients with hay fever during natural allergen exposure.. J Allergy Clin Immunol.

[PDF_00596] Weeke B. (1973). Rocket immunoelectrophoresis.. Scand J Immunol Suppl.

